# Solidification Enhancement in a Multi-Tube Latent Heat Storage System for Efficient and Economical Production: Effect of Number, Position and Temperature of the Tubes

**DOI:** 10.3390/nano11123211

**Published:** 2021-11-26

**Authors:** Min Li, Jasim M. Mahdi, Hayder I. Mohammed, Dmitry Olegovich Bokov, Mustafa Z. Mahmoud, Ali Naghizadeh, Pouyan Talebizadehsardari, Wahiba Yaïci

**Affiliations:** 1 Digital Economy Academy, Yango University, Fuzhou 350015, China; look87@126.com; 2 Department of Energy Engineering, University of Baghdad, Baghdad 10071, Iraq; jasim@siu.edu; 3 Department of Physics, College of Education, University of Garmian, Kurdistan, Kalar 46021, Iraq; hayder.i.mohammad@garmian.edu.krd; 4 Institute of Pharmacy, Sechenov First Moscow State Medical University, 8 Trubetskaya St., Bldg. 2, 119991 Moscow, Russia; fmmsu@mail.ru; 5 Radiology and Medical Imaging Department, College of Applied Medical Sciences, Prince Sattam bin Abdulaziz University, Al-Kharj 16244, Saudi Arabia; m.alhassen@psau.edu.sa; 6 Faculty of Health, University of Canberra, Canberra 2601, Australia; 7 Faculty of Mechanical Engineering, Babol University of Technology, Babol 4714873113, Iran; ali.naghizadeh2412@gmail.com; 8 Centre for Sustainable Energy Use in Food Chains, Institute of Energy Futures, Brunel University London, Uxbridge UB8 3PH, UK; 9 Canmet ENERGY Research Centre, Natural Resources Canada, 1 Haanel Drive, Ottawa, ON K1A 1M1, Canada

**Keywords:** natural convection, phase change material, tubes’ arrangement, thermal energy storage, solidification, multi-tubes heat exchanger

## Abstract

Thermal energy storage is an important component in energy units to decrease the gap between energy supply and demand. Free convection and the locations of the tubes carrying the heat-transfer fluid (HTF) have a significant influence on both the energy discharging potential and the buoyancy effect during the solidification mode. In the present study, the impact of the tube position was examined during the discharging process. Liquid-fraction evolution and energy removal rate with thermo-fluid contour profiles were used to examine the performance of the unit. Heat exchanger tubes are proposed with different numbers and positions in the unit for various cases including uniform and non-uniform tubes distribution. The results show that moving the HTF tubes to medium positions along the vertical direction is relatively better for enhancing the solidification of PCM with multiple HTF tubes. Repositioning of the HTF tubes on the left side of the unit can slightly improve the heat removal rate by about 0.2 in the case of p5-u-1 and decreases by 1.6% in the case of p5-u-2. It was found also that increasing the distance between the tubes in the vertical direction has a detrimental effect on the PCM solidification mode. Replacing the HTF tubes on the left side of the unit negatively reduces the heat removal rate by about 1.2 and 4.4%, respectively. Further, decreasing the HTF temperature from 15 °C to 10 and 5 °C can increase the heat removal rate by around 7 and 16%, respectively. This paper indicates that the specific concern to the HTF tube arrangement should be made to improve the discharging process attending free convection impact in phase change heat storage.

## 1. Introduction

Improving the performance of energy technology is a promising solution to replace conventional sources with renewable technology. Recently, energy storage units have achieved a level with a high influence and application in utilized technology [1]. Energy storage systems can be helpful by saving waste energy and releasing it to reduce the consumption of the national grid [2,3]. Thermal energy storage (TES) is a type of energy storage which stores and release heat during the melting and solidification period of the applied phase change materials. Thermochemical, sensible, and latent heat thermal energy storage are various types of TES systems. Latent heat storage (LHS) operates the phase change processes within a fixed temperature (phase change point). It has advantages of high storage density (because of considerable latent heat of fusion), isothermal nature of the storage method, easy control, and applicability for multi-cycles. Accordingly, LHS is considered the best technique of TES. Phase change materials (PCMs) can apply in the TES for both latent and sensible methods and they are important for energy management [3]. PCM undergoes the charging and discharging processes to melt and solidify at a specific temperature. The main dilemma of utilizing the PCM is the weak thermal conductivity, which reduces the performance of the system. Thus, various techniques were applied to enhance the thermal conductivity of the TES [4]. These techniques include utilizing fins or extended surfaces [5,6,7,8,9], adapting the system body and structure [10], metal foam [11,12,13,14,15,16], heating and cooling channel [16,17,18,19], treating with high conducting particles [20,21], multiple-PCMs [22,23,24], nanoparticles [16,25], improving the convection heat transfer [26,27,28], and using the combinations of different methods [29,30,31,32]. Multi heat transfer fluid channels could be used to improve the free convection of the system, which is a similar effect on the weight and the cost of the entire system. The functional nanofluid containing nanosized PCM particles are also another type of PCM materials [33,34,35]. The applications of PCM in various areas, such as the solar cooling and space industry, photovoltaic units, waste heat recovery units, preservation of food and pharmaceutical products, have been considered in the last decade [36].

In the case of engineering designs, there are couple categories of the heat exchanger: shell and tube units [31,37] and triple homocentric channels [38,39] system. More than 70% of research on LHTES units concentrate on the shell-and-tube mode, due to its channel design and lower heat loss [40]. The cylindrical shell-and-tube system has shown its feature regarding the heat transfer rate, under similar situations. The shell and tube thermal exchanger treated with fins or extended surfaces is considered an ideal case due to the high heat transfer efficiency, simple design and easier connection to applications. Such a system depends on the type and the location of the PCM, and the number and places of heat transfer fluid (HTF) channels, which are likewise classified into various forms: pipe, cylinder, and multi-tube [2]. In a cavity form, Ghalambaz et al. [41] investigated the flow and heat transfer of PCMs in the presence of a magnetic field. The results showed that a magnetic field can control the melting behavior by influencing the natural convection in the PCM molten regions.

In the tube form, the HTF moves on the external wall, while the PCM exists in the tube and the HTF. The cylinder form considers as the best shell-tube design [42], in which the HTF flows in the channel surrounded by the PCM. Pakalka et al. [43] investigated the thermal free convection in the fins combined heat exchanger. They stated that the thermal convection coefficient significantly improves in both experimental and theoretical procedures. Mahdi et al. [15] studied the response of the heat transfer in the shell-and-tube TES involving multi-segment with separate PCMs of various phase change temperatures. They found that utilizing multi-segment improves the phase change velocity by 94%. Rathod and Banerjee [44] explored the phase change behavior in the shell and tube. They found that the velocity of the phase change process enhances by 25 and 44% with and without using fins. Lohrasbi et al. [45] confirmed that using fin and tube in the vertical shell and tube system improves the melting rate more than 3.3 times over the finless case. Esapour et al. [46] numerically investigated the best number and diameter of the multiple channel units. They found that the charging time reduces by 29% when four heating channels are utilized compared with a single channel, and the tube placed at the lower part of the heat exchanger improves the efficiency of the entire system. Rabienataj Darzi et al. [47] stated that the phase change time reduces when the fins are utilized in the shell and tube system. They quantified that the charging and discharging time reduces by 110% and 203% when the number of the utilized fins increases from four to twenty. Khan et al. [48] innovate a new design of shell and tube joint with extended surface fins. They stated that increasing the inlet temperature from 323 to 343 K significantly reduce the phase change time and total enthalpy by 69% and 18%, respectively. 

Numerous critical parameters were stated for the common geometries while investigating the phase change process inside these systems. Thermal convection and conduction manage the heat transfer and the phase change process in the domain. The cells affect the phase change process at the base part of the solid PCM in the domain and the free convection controls the upper part [21 Mohagh] [4,49,50,51]. The earliest work investigated the phase change process has been attained by Tao [52]. He developed a model to track the boundary shifting process during the phase change method. Gortych et al. [53] investigated the discharging process of the PCM in the horizontal tube experimentally and numerically. They proposed a fixed wall temperature and typical values of the natural convection were detected. Abdollahzadeh and Esmaeilpour [54] assessed the effect of the nanofluid on the phase change process of the PCM in the wavy wall domain. They found that the nanofluid and the shape effect of the system are highly affecting the performance of the TES. Shahsavar et al. [55] studied the effect of the system shape and the metal foam on the TES. They detected that the enlarging heat transfer surface area caused by the metal foam is significantly affecting the heat transfer efficiency of the solidification process. The metal foam impact on the phase change was also addressed in some recent studies [56,57]. Choi and Kim [58] evaluated the circular fins for the discharging improvement in the TES. They showed that the fins improve the thermal coefficient by three times over the no-fins case. Wang et al. [59] numerically examined the solidification mode in a zigzag shape heat exchanger. A significant effect on the thermal efficiency was observed by increasing the average velocity of the HTF. Sardari et al. [10] analyzed the modified zigzag configuration of the LHS. They confirmed that the unit with the zigzag angle of 60° increases the storage time threefold over the time of the situation with a 30° zigzag angle.

Many researchers have studied the optimization of the various dependent parameters to reach the best efficiency, lower cost, and desired size. Bazai et al. [17] analyzed the heat transfer of the annular tubes TES unit. They revealed the optimum values of the tubes’ cross-section area angular location. They detected that the larger angle of the channel created a faster charging rate, the charging time decreased by 61%, and the efficiency improved by 26% when the aspect ratio (hydraulic diameter to the length of the channel) was 1/3 compared with the aspect ratio of 1. Talebzadehsardari et al. [16] examined the best configuration and position of airflow channels on the solidification process of a combined porous medium-PCM system. They found that the discharging rate increased by 57%, and the temperature difference between the two ends of the HTF tube improved threefold compared with the system with the conventional HTF tube. Li et al. [60] mathematically detected the optimization of the packed-bed LHS with cascaded PCM capsules with the effect of the limitation of outlet threshold temperature. They revealed that the actual usage rate can reach almost double as high as that in non-cascaded PCM-TES. Liang et al. [40] analyzed the best case for shell-and-tube TES. The flow pattern considerably enhanced the phase change rate by 50 times and increased the efficiency at the applied PCM volume ratio from 0.2–0.8 to 0.6–0.9 at laminar flow cases.

The review of the previous works indicated that many parameters have a considerable influence on the discharging process. Although extensive research was carried out on the functionality of the multi-tube heat exchanger as an effective PCM containment design, no studies exist that adequately cover the influencing parameters such as the number, position, and temperature of the HTF tubes during the energy discharging (solidification) mode. A significant improvement would be achieved in the entire performance with no additional cost, as no additional material is used in the fabrication of the storage system. Therefore, analyzing and studying the best location and number of the multi-tubes delivers well information and knowledge in this field. Thus, the novel idea of this work is designed to scientifically evaluate and improve the performance of a shell-and-tube LHTES to increase the discharging rate and enhance the thermal rate released from the PCM. The distance between the tubes and the top and bottom walls was optimized to gain a higher discharging rate. Free convection influences the whole discharging process, and it is described implicitly in the manuscript. The assessment was attained by studying the liquid fraction (LF), the discharging rate, the contours of the phases, and the temperature. The purpose of this work was to push the HTF tubes’ position through the PCM domain to achieve the best position and take full advantage of natural convection flows to increase the solidification rate.

## 2. System Description

**Figure 1:**
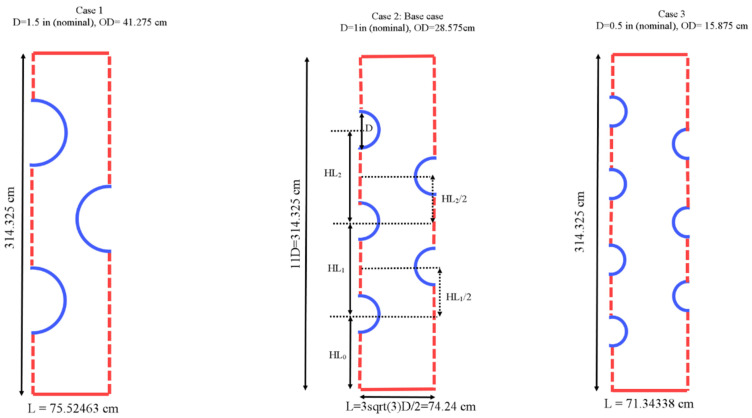
The schematic of the proposed multitube heat exchanger.

The proposed system studied in this paper is a multitube shell and tube heat exchanger incorporating different tube numbers of 3, 5, and 5 in a rectangular PCM container which is evaluated during the solidification process. Figure 1 illustrates the schematic of the proposed heat exchanger in a symmetrical condition from both left and right sides that could be extended by repeating the pattern in the horizontal direction. This assumption assumes a long width for the heat exchanger. The heat exchanger’s upper and lower walls are insulated with a no-slip boundary condition. Different tubes’ wall temperatures of 5, 10, and 15 °C are considered while the initial temperature of the PCM is equal to 50 °C. The gravity is considered in the vertical direction toward the bottom of the heat exchanger. It should be noted that the system is studied in a two-dimensional condition due to having no changes in the z-direction with the assumption of considering a long length and ignoring the wall effects.

Three different diameters are considered for the tubes in different systems while the total area of the tubes is considered constant. Thus, the masses of the PCM in the systems are similar. The nominal diameters of the tube (D) are 0.5 in, 1 in, and 1.5 in and thus the outer diameters of tubes are 15.875, 28.575, and 41.275 mm. Note that the tubes are selected from the standard sizes of copper tubes in heat exchangers. The height of the shell is assumed to be 314.325 mm, and the width of each repeated section is considered to be 71.344 mm which are selected based on the system with five tubes as shown. It is assumed that the distance between two pipes that are next to each other on the left wall and the adjacent pipe on the right wall is constant. In addition, the tubes on the right-hand side of the shell are located at the centerline of the two adjacent tubes on the left-hand sidewall. In other words, the centers of these pipes generate an equilateral triangle. The reason for using a staggered array of the pipes is a better and more uniform distribution of the heat sinks in the heat exchangers considering the constant area for the tubes. Thus, during the solidification process, a heat sink can fill the gap between the two adjacent pipes in the vertical direction which can help improve the solidification rates of the heat exchanger. For the best configuration, the position of the tubes (distance from the bottom) is evaluated to study the effect of natural convection and find the best arrangement to have the highest solidification rate. 

RT-35 from Rubitherm company is used as the PCM in this study. The properties of RT35 are presented in Table 1. 

## 3. Mathematical Modeling

The heat recovery from PCMs typically takes place during their solidification mode via thermal conduction in the solid component and via the conduction and natural convection in the liquid component of the PCM. This, in turn, causes a buoyancy-driven flow in the PCM’s liquid component when temperature gradients existing between the different PCM layers are sufficiently efficient or effective to compete for the gravity effect [62]. To simplify the mathematical formulation for the PCM solidification problem, the following assumptions were applied: (1) PCM is initially in the liquid phase, and its liquid flow is timewise and incompressible; (2) viscous dissipations are insignificant and can be neglected; (3) the change in temperature of the HTF during operation is negligible, (4) no-slip conditions for fluid velocities at the solid boundaries; (5) all thermal properties of the PCM are temperature-independent except density in the momentum equations; and (6) variation of temperature is accounted for by employing Boussinesq approximation. The governing equations based on the aforementioned assumptions for 2D laminar and Newtonian fluid flow are provided as follows built on the enthalpy-porosity technique defined by Brent et al. [63]:

(1) $$\frac{\partial \rho }{\partial t}+\nabla \cdot \rho \vec{V}=0$$

(2) $$\frac{\partial \left(\rho u\right)}{\partial t}+\nabla \cdot \left(\rho u\vec{V}\right)=-\frac{\partial P}{\partial x}+\nabla \cdot \left(\mu \nabla u\right)-\frac{A_{m}\left(1-\beta^{2}\right)}{\lambda^{3}+0.001}u$$

(3) $$\frac{\partial \left(\rho v\right)}{\partial t}+\nabla \cdot \left(\rho v\vec{V}\right)=-\frac{\partial P}{\partial y}+\nabla \cdot \left(\mu \nabla v\right)-\rho g\beta \left(T-T_{ref}\right)-\frac{A_{m}\left(1-\beta^{2}\right)}{\lambda^{3}+0.001}v$$

(4) $$\rho C_{p}\frac{\partial T}{\partial t}+\rho C_{p}\nabla \left(\vec{V}T\right)=\nabla \left(k\nabla T\right)-\left[\nabla \left(\rho \vec{V}\lambda L_{f}\right)+\frac{\partial \rho \lambda L_{f}}{\partial t}\right]$$

where $A_{m}$ is the mushy constant which is selected as 10^5^ followings [42]. The symbol λ shows the liquid volume fraction (melted fraction of PCM in an element) defined as:

(5) $$\lambda =\frac{\Delta H}{L_{f}}=\left\{\begin{array}{c} 0ifT<T_{S}\\ \frac{\left(T-T_{S}\right)}{\left(T_{L}-T_{S}\right)}ifT_{S}<T<T_{L}\\ 1ifT>T_{L} \end{array}\right\}\;$$

where the total enthalpy of an element is the combination of latent heat (Δ*H*) and sensitive enthalpy (*h*) as H= ΔH+h. Thus, the total enthalpy could be computed by the integration of *H* over the computational domain. 

The solidification or discharging rate $\dot{Q}$ is introduced as [64]:

(6) $$\dot{Q}=\frac{Q}{t_{m}}=\frac{m\left(\int_{S}^{\;}C_{p}dT+L_{f}+\int_{L}^{\;}C_{p}dT\right)}{t_{m}},$$

where $t_{m}$ is the solidifying time and m is the mass of PCM. 

## 4. Numerical Process

The governing equations are solved numerically using ANSYS-FLUENT software. The SIMPLE algorithm with PRESTO and QUICK methods are employed to discretize the momentum and energy equations, respectively. The convergence criteria are set to 10-4 for continuity and 10-6 for the momentum and energy equations. Furthermore, it should be noted that different grids and sizes of time-step are also evaluated to have the results independent from the grid and time step size. 

**Figure 2:**
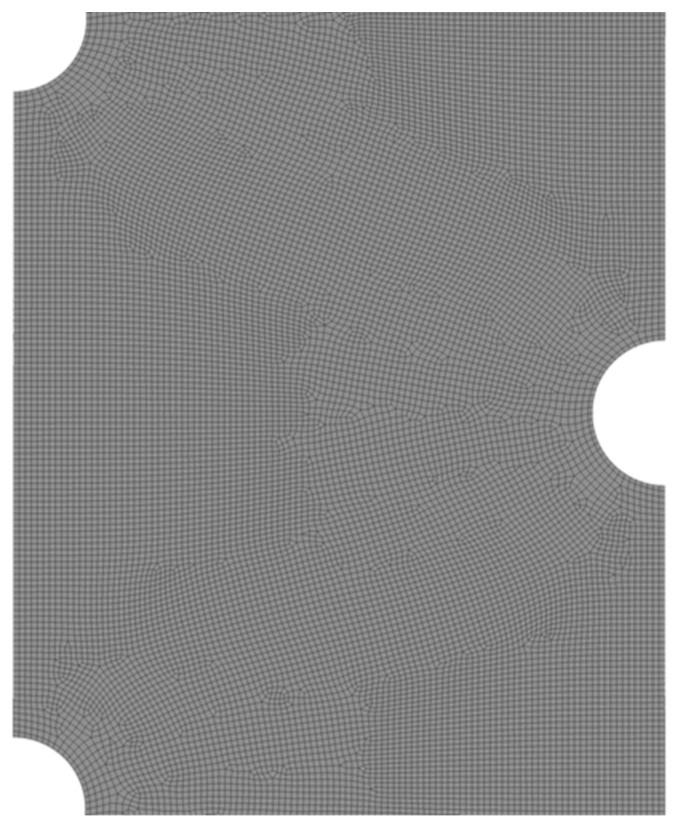
The mesh with 52,682 nodes in the middle of the domain.

The solidification rate is evaluated as the criteria to find the mesh independent from the number of cells and size of the time step. Different node numbers of 35,122, 52,682, and 10,5364 are evaluated considering the time step size of 0.2 for the case with seven tubes. The results are presented in Table 2. As shown, the solidification rates for the meshes with 52,682 and 10,5364 nodes are almost similar (the difference is less than 0.5%) and thus the mesh with 52,682 nodes is selected for further analysis. Figure 2 shows the mesh of the selected case for the middle part of the domain. Different time step sizes of 0.1, 0.2, and 0.4 s are also studied to find the results independent from the size of the time step for the selected mesh presented in Table 2 showing almost similar solidification rates and therefore, the size of the time step is also selected equal to 0.2 s. 

**Figure 3:**
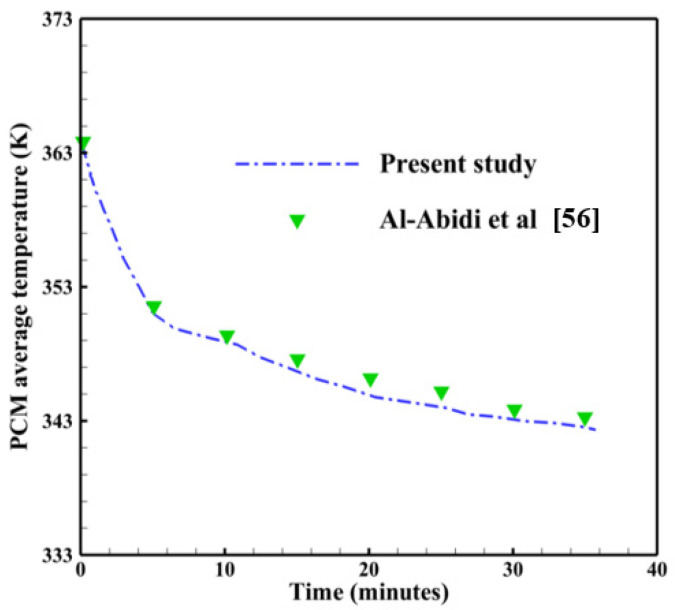
Code verification during the solidification process using the experimental study of Al-Abidi et al. [65].

To validate the accuracy during the solidification process, the present study is compared with the experimental work of Al-Abidi et al. [65]. They investigated the PCM temperature variation with the presence of longitudinal fins. The containment unit was a triplex-tube heat exchanger, which was made up of three aluminum tubes that were concentrically placed in a horizontal position. The heat-transfer fluid (water) is to be injected into the interior tube and the exterior tube from the inside to provide the necessary heating or cooling effect. PCM, which was paraffin RT82, was sandwiched in the annular area between the interior and middle tubes. The results of the PCM average temperature found in this work and those found in Al-Abidi et al. [65] are shown in Table 3. The maximum error calculated is 0.029%, which could be ignorable. As observed in Figure 3, the comparison of the PCM average temperature of the present study is in good agreement with this experimental study of Al-Abidi et al. [65] showing the accuracy of the present model for the solidification process.

## 5. Results and Discussion

Due to the temperature variation which exists between the different PCM layers during solidification, heat removal occurs solely by conduction in solid PCM while it occurs through conduction and natural convection as well in liquid PCM. The buoyancy-driven flow which is produced in the liquid portion of PCM as a result of the density difference between the solid and liquid PCM layers drives the process of natural convection evolution. The heated liquid of PCM typically has a tendency to migrate upward under the effect of buoyancy, and because the upper sections of the domain are always subject to warmer heat currents, local convection is promoted to serve as an additional supply of heat diffusion in these parts of the unit. Therefore, energy storage units should be designed properly to gain maximum benefits from natural convection and thermal conduction as a combination. 

Applying the simulation model described earlier in this study, several runs were carried out to optimize the design of the PCM-based storage unit with multiple tubes transporting the heat-transfer fluid (HTF). The effects of employing different tube arrangements on the thermofluidic performance of the thermal storage unit during the energy discharging mode are addressed and demonstrated in detail. Four geometrical design variables, designated as HL0, HL1, HL2, and HL3, were assessed as three different arrangements (P3, P5, and P7) with the dimensions of each arrangement are reported in Figure 2. Each of these arrangements has three, five, or seven tubes, depending on the total heat transfer area being required. The area of heat transfer was preassigned to be the same in all arrangements to enable meaningful comparisons to be made between the various scenarios under consideration. 

The results of this study are organized into four sections. In Section 5.1, the solidification behavior of PCM in a multi-tube storage unit is explored in further detail. Later in Section 5.2 and Section 5.3, the impacts of tube distribution on the right and left sides of the storage unit are optimized and discussed. Finally, the impacts of the HTF inlet temperature on PCM solidification are examined in Section 5.4.

### 5.1. Impact of Varying the Number of HTF Tubes

**Figure 4:**
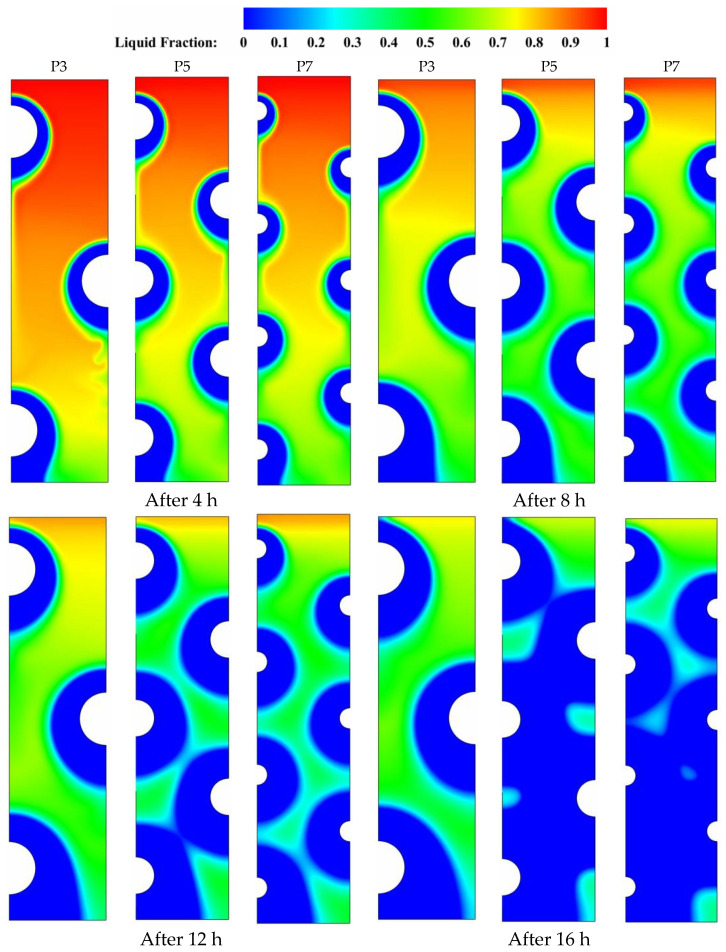
Contours of the PCM liquid-fraction progression for three different HTF-tube arrangements of P3, P5, and P7 over multiple solidification durations.

**Figure 5:**
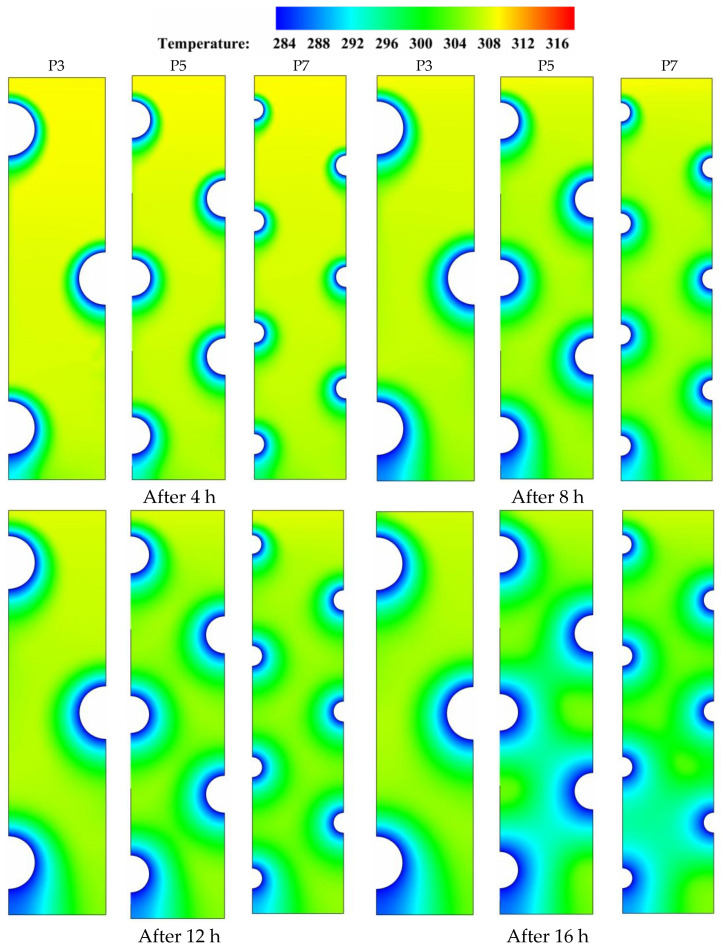
Contours of the PCM temperature distribution for three different HTF-tube arrangements of P3, P5, and P7 over multiple solidification durations.

**Figure 6:**
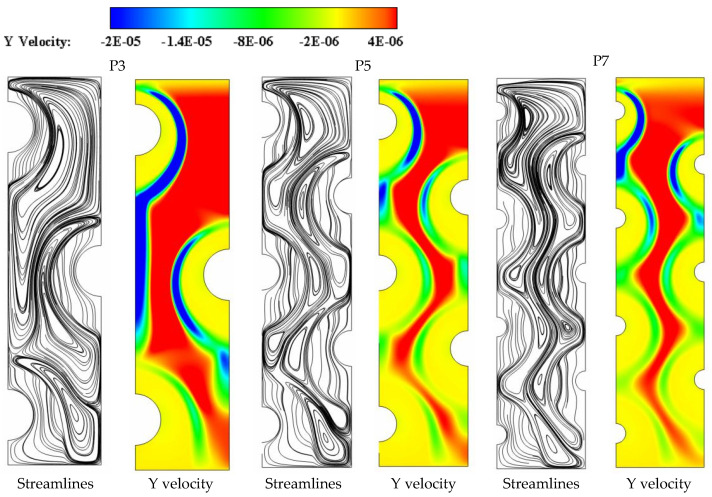
The velocity field and streamlines for three different HTF-tube arrangements of P3, P5, and P7 after 8 h of solidification.

**Figure 7:**
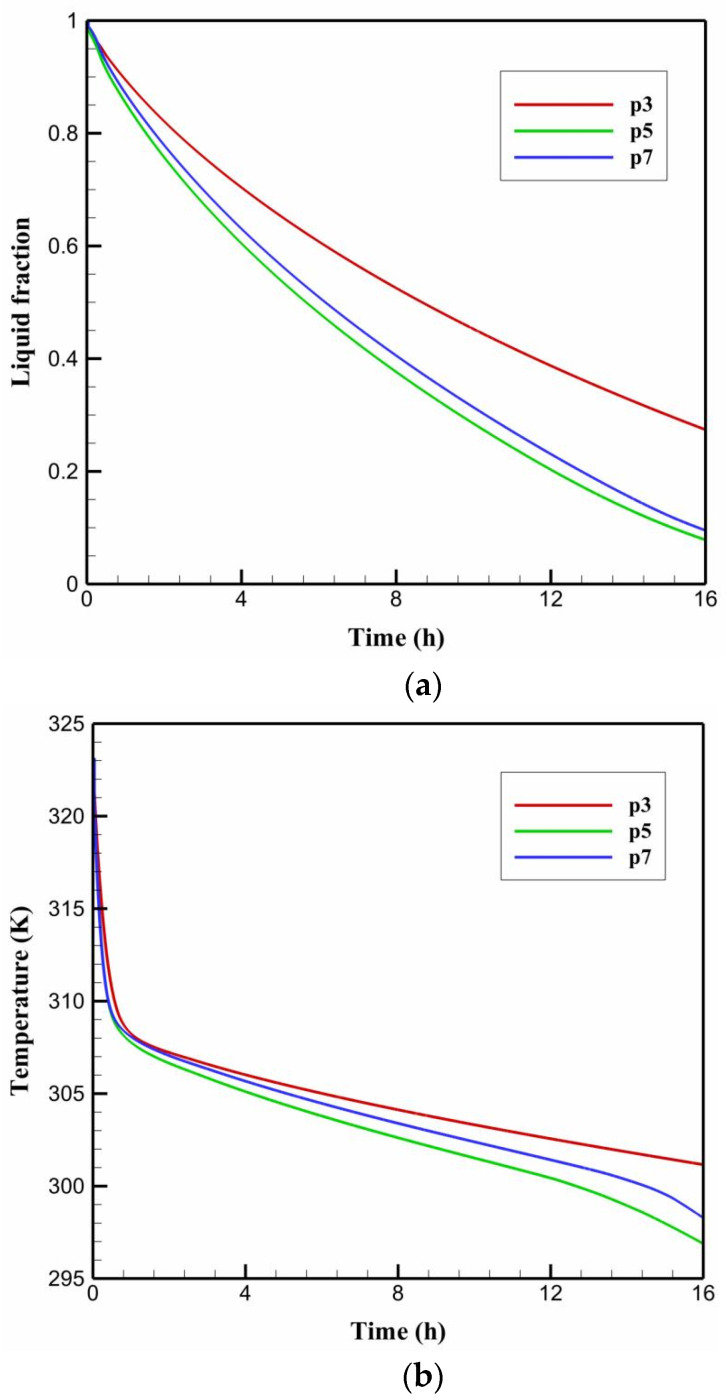
The time-wise variation of (**a**) liquid-fraction profile, and (**b**) average temperature for the PCM solidification with tube arrangements of P3, P5, and P7.

**Figure 8:**
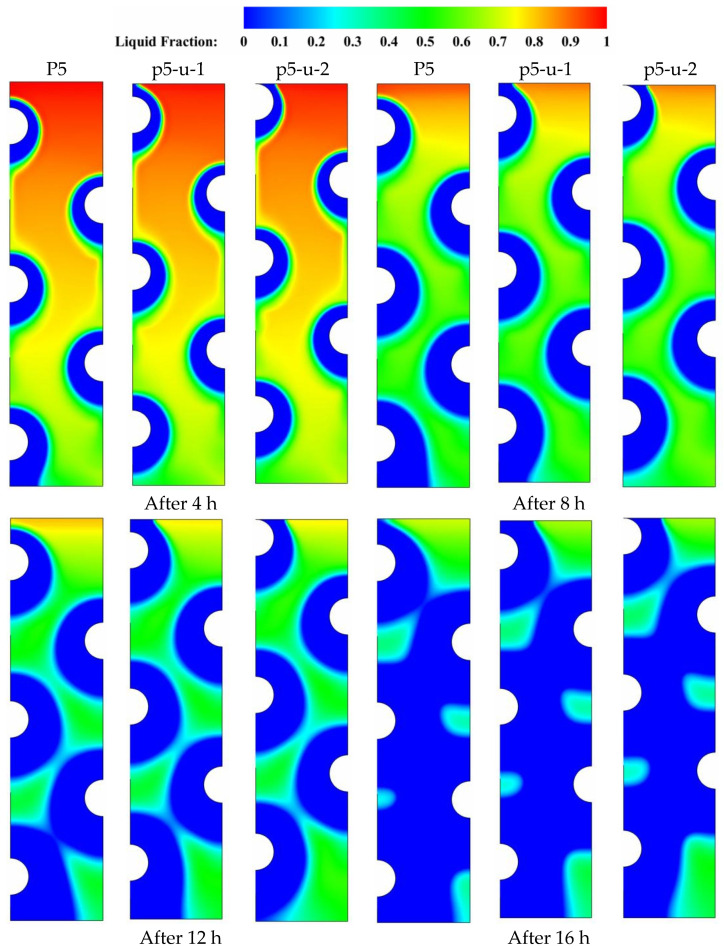
Contours of the PCM liquid-fraction progression for three different HTF-tube arrangements of P5, p5-u-1, and p5-u-2 over multiple solidification durations.

To explore the beneficial arrangement of HTF tubes to facilitate better solidification behavior in the PCM-based storage unit, three distinct tube arrangements, consisting of 3, 5, and 7 tubes, are examined concerning the PCM solidifying properties. Figure 4, Figure 5, Figure 6, Figure 7 and Figure 8 show the effects of the tube arrangement on the temporal evolution of the liquid-fraction and temperature distribution, as well as the velocity field and streamlines, during four distinct solidification periods (t = 48, 12, and 16 h). During the initial duration (t = 4 h) of solidification, the latent heat of PCM begins to release to the low-temperature HTF, which is followed by the formation of a solidified layer (blue area) at the surface of the HTF tube and proceeds from there to invade the other parts of the PCM domain. As the heat release from PCM to the HTF tubes continues to rise, the thickness of the solidified PCM layers on the HTF tube surfaces increases, and the impact of natural convection gets further minimized. 

It is worth mentioning that natural convection initially dominates the heat release process. However, as the solidified layer increases in size, thermal conduction becomes the primary mode of heat transfer. This results in a reduction in the convection-assisted circulation of liquid PCM throughout the domain as the thickness of solidified PCM layer around the HTF tube continues to grow up. This can be seen from Table 4 and Figure 4 when comparing the sizes of the blue zone, which denote the solidified PCM layers at later durations (t = 8, 12, and 16 h), to those in the base duration (t = 4 h). It can also be seen from Figure 4 that the solidification evolution history witnesses the greatest improvement in the case of P5 compared to other cases of P3 and P7. During the final duration (t = 16 h), solidification seems to occupy the major area of the domain in the case of P5 (the blue area) much better than that in the other cases. Therefore, increasing the number of HTF tubes used does not always result in improved PCM solidification. It would even have a detrimental impact on the potential for solidification enhancement in the liquid zones. This can be due to the positive role of natural convection in the solidifying process, particularly in the upper sections of the domain. Due to the high flow resistance generated by the high number of HTF tubes used, natural convection-assisted circulation of the liquid PCM layers is significantly reduced, resulting in slower discharging rates for the thermal storage module.

Figure 5 depicts the temperature distribution on a range of time durations for various HTF-tube configurations that were investigated. Temperature gradients are established across the PCM domain when heat is initially transmitted between the tubes currying the low-temperature HTF and the adjacent solidifying layers (shown in green) that surround the tube zones at the early periods of solidification (t = 4 to 8 h). This results in the advancement of the role played by thermal conduction, which, once a sufficient amount of time has elapsed, becomes able to dominate the whole heat release process and helps in the formation of bigger solidifying layers over the major PCM domain. During the later durations (t = 12 to 16 h), a significant variation in the color of isotherms is seen as a sign of the initiation of an effective role by natural convection. At this time, both natural convection and thermal conduction have an impact on the PCM temperature distribution, but conduction continues to play the dominating role as long as the isotherms preserve the uniformity in temperature distribution. For example, comparing the isotherms at t = 16 h in the case of five tubes (P5) to the base case of three tubes (P3) shows that there is a discoloration in the case of P3 due to the higher temperature gradients generated by the presence of convective flow in the upper and middle portions of the PCM domain. Furthermore, the major domain is seen to be more unified in color when the number of tubes increases from five to seven, as a result of the weakening of the buoyancy effect caused by the high flow resistance associated with the higher number of HTF tubes. Generally speaking, the use of a high number of HTF tubes helps conduction to dominate the PCM solidification process over natural convection due to the high flow resistance generated by the tube arrangement structure, which in turn assists in more uniform PCM temperature distribution.

The contours for streamlines and velocity field that are shown in Figure 6 confirm that during the duration (t = 8 h), the increase of tube number from the base case of three tubes (P3) to five tubes (P5) or seven tubes (P7) could further weaken the heat transport by convection in the liquid PCM. For example, increasing the number of HTF tubes from P3 to P7 drives the velocity field to indicate slower convection-assisted recirculation of liquid PCM with noticeable shrink in the red layers that represent the zones of high velocities. Meanwhile, certain recirculation cells form in the spaces between the HTF tube zones, particularly in the upper sections of the domain. This is because the buoyancy effect, which originates from the density difference between the heated liquid PCM and the cold solid PCM, is stronger than the gravity effect and therefore, drives the liquid PCM to flow with generating a group of recirculation cells. Increasing the number of HTF boosts developing more but smaller recirculation cells as can be seen in Figure 6 when comparing the cases of P7 and P5 to the base case of P3.

Figure 7a,b depict the impact of varying the number of HTF tubes on the time-wise evolution of the liquid-fraction and the average temperature profiles, respectively. The data from Figure 7 indicates that increasing the number of tubes from the case P3 of three tubes to the case P5 of five tubes results in the greatest improvement in the PCM liquid-fraction and temperature behaviors. It is observed that the liquid-fraction and average temperature behavior curves in the case of P5 retain the lowest values, resulting in a shorter time required for the discharging time as compared to the other cases studied. It should be noted that, despite the fact that the number of tubes employed in the case of P7 is higher in terms of the tube heat transfer area, the discharge time is still significantly longer than that in the case of P5. These results indicate that if an optimal number of HTF tubes is employed, natural convection will be able to contribute more effectively to the heat removal process. This, in turn, aids in the reduction of flow resistance, which results in a lower PCM temperature and faster solidification rates.

### 5.2. Impact of Varying the Tube Distribution on the Left Side 

To gain the benefits of natural convection’s nonuniformity in the vertical direction, energy storage units that consist of multiple HTF tubes must be designed in a manner that the tube arrangement can maximize their storage capabilities. In this section, three tube arrangements with the same number of tubes but different tube distributions along the left vertical direction are studied and discussed. All three tube arrangements have the same vertical distance between the centers of tubes. P5 serves as the reference case against which two additional cases, p5-u-1 and p5-u-2, are compared and discussed. P5-u-1 and P5-u-2 represent the cases in which the HTF tubes are moved to the medium and top locations, respectively, along the vertical direction of the storage unit. Figure 8 depicts the liquid-fraction contour distributions over four different time durations (t = 4, 8, 12, and 16 h) for the three tube arrangements under consideration. During the early durations (t = 4 to 8 h), natural convection takes over as the predominant heat transport mode, allowing for a slight delay in the solidification of the PCM at the upper sections of the unit. Meanwhile, the size of the solidified PCM layers (the blue zone) is greatly influenced by the sort of tube configuration that is employed in the specific case. When the size of solidified PCM is compared between the three tube distribution cases, the size of the solidified PCM layer gets smaller as the tube arrangement moves away from the base case of P-5. This is true for all time durations. As can be observed in Figure 8, the solidification evolution experienced the greatest delay in the case of top distribution (P7-u-2). Accordingly, repositioning the HTF tubes from their medium positions would have a negative impact on the potential for solidification propagation in the liquid zones. As a result, solidification takes a longer time to complete, particularly in the case of top tube arrangement (P7-u-1). The simulation results show that the values of liquid fraction after 16 hr of solidification are 0.078, 0.075, and 0.090 for the cases of P5, P5-u-1, and P5-u-2, respectively. This implies that changing the tube distribution along the vertical direction would not bring a big improvement to the solidification behavior of PCM. However, moving the HTF tubes to medium positions along the vertical direction is relatively better for enhancing the solidification of PCM with multiple HTF tubes. 

**Figure 9:**
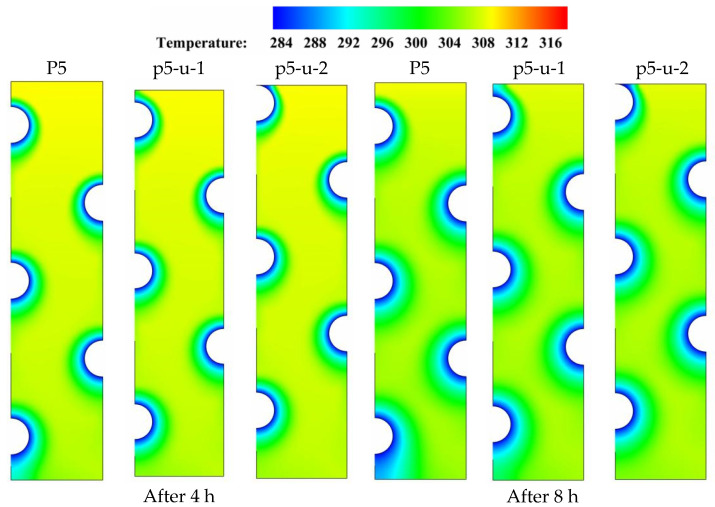

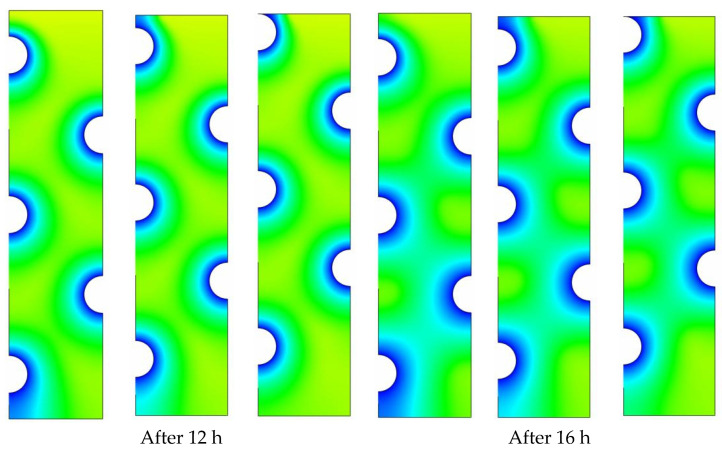
Contours of the PCM temperature distribution for three different HTF-tube arrangements of P5, p5-u-1, and p5-u-2 over multiple solidification durations.

Figure 9 displays the temperature contour distributions over four distinct time durations (t = 4, 8, 12, and 16 h) for each of the three tube arrangements P5, P5-u-1, and P5-u-2 under discussion. As time progressed, a significant variation in the color of the isotherms is seen as the tube positions are moved from their original positions in the base case of P5. Comparing the isotherms of the middle tube arrangement (P5-u-1) to the base case (P5) reveals that the color of the isotherms deviates from uniformity in the upper part, due to the gradual temperature gradient generated by the presence of buoyancy-driven flow in the upper parts of the domain. In all time durations, the presence of this discoloration indicates that natural convection has played a role in the overall heat removal process, which is beneficial to the solidification progression. However, this convection contribution is negatively affected when the HTF tubes are moved to the top locations in the case of upper tube distribution (P5-u-2). This is because the local flow resistance generated by the presence of HTF tubes increases with moving the tubes to upper locations, resulting in a reduction in the convection-assisted circulation of liquid PCM. As a result, when comparing the solidification behavior of PCM to that of the base case of P5, moving the HTF tubes along the left vertical direction does not bring substantial improvements to the overall solidification process of PCM. This would be due to the dominating role of conduction during the entire discharging mode. Data from Table 5 shows that the PCM can solidify at an energy removal rate of 99.42 W in the base case of P5, but this rate increases to 99.54 and decreases to 97.94 W when the HTF tubes are moved vertically on the left side in the cases of p5-u-1 and p5-u-2, respectively. Therefore, repositioning of the HTF tubes on the left side of the unit can slightly improve the heat removal rate by about 0.2 in the case of p5-u-1 and decreases by 1.6% in the case of p5-u-2. 

### 5.3. Impact of Varying the Tube Distribution on the Right Side

**Figure 10:**
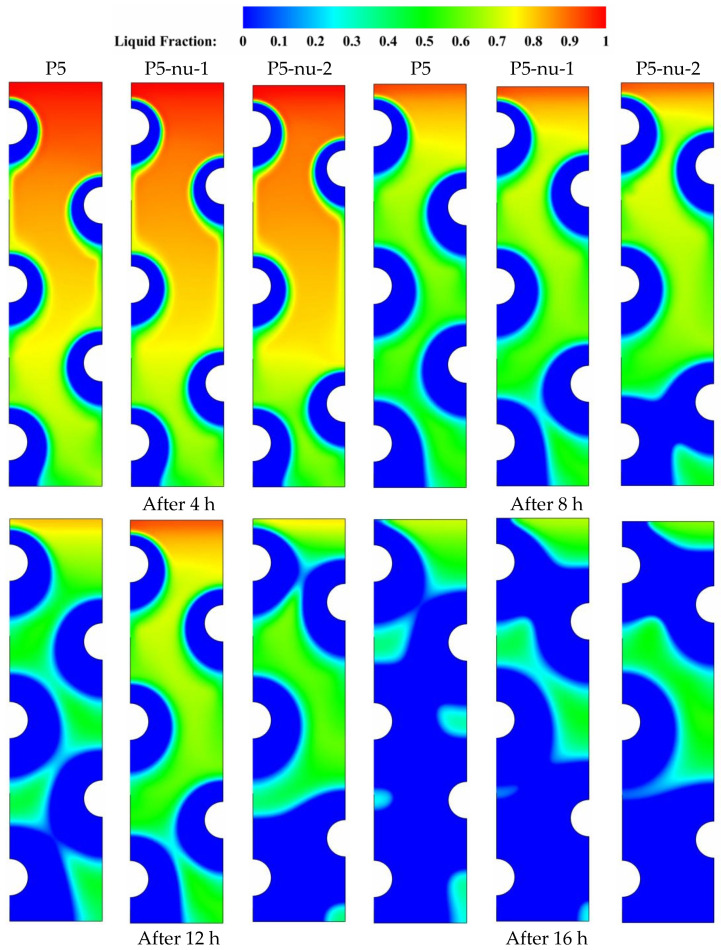
Contours of the PCM liquid-fraction progression for three different HTF-tube arrangements of P5, p5-nu-1, and p5-nu-2 over multiple solidification durations.

This section examines the impact of varying the distance between the HTF tubes on the right side on the potential of solidification enhancement with multiple HTF tubes. Three different tube arrangements having the same number of tubes along the right vertical direction are compared and discussed in Figure 10. The case of P5 is the reference case to which the two additional cases (P5-nu-1 and P5-nu-2) are compared and analyzed. P5-nu-1 and P5-nu-2 represent the cases where the distance between the two HTF tubes on the right side gradually increases in the vertical direction. During the initial durations (t = 4 to 8 h), natural convection becomes an effective heat transfer mechanism which triggers a slight delay in solidifying the PCM in the upper regions of the domain. The size of the solidified PCM layers (the blue zone) is highly dependent on the type of tube arrangement used. It can be observed from Figure 8 that the size of the solidified PCM layer decreases when the tube arrangement departs from the reference arrangement of P5. It can also be seen from Figure 10 that the case of largely spaced tubes (P7-u-2) exhibits the longest delay in the evolution of solidification. Therefore, repositioning the HTF tubes from their medium placements negatively affects the potential for solidification propagation in the liquid zones. As a result, solidification takes a longer time to complete, especially in the case of largely spaced tubes (P7-nu-2). Table 6 indicates that after 16 h of solidification, the results indicate that the liquid fraction is 0.014, 0.011, and 0.11 for the cases of P5, P5-nu-1, and P5-nu-2, respectively. This means that increasing the distance between the tubes in the vertical direction has a detrimental effect on the PCM solidification mode.

**Figure 11:**
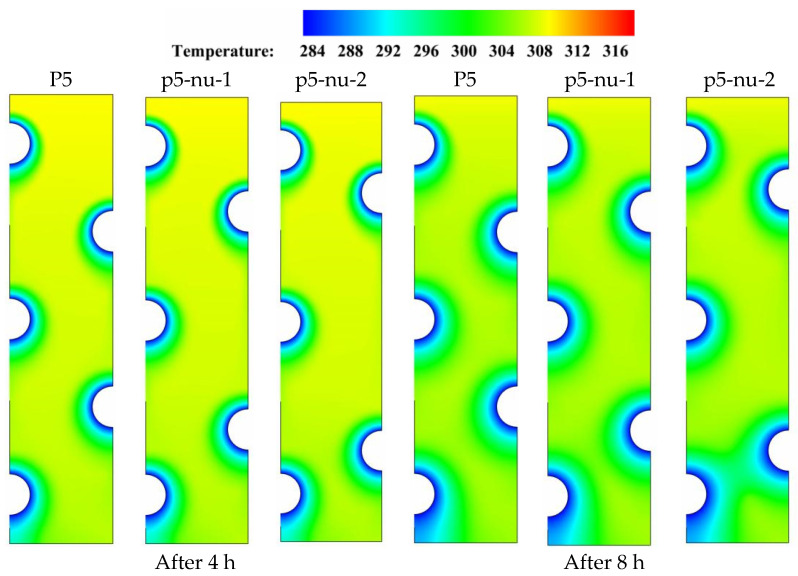

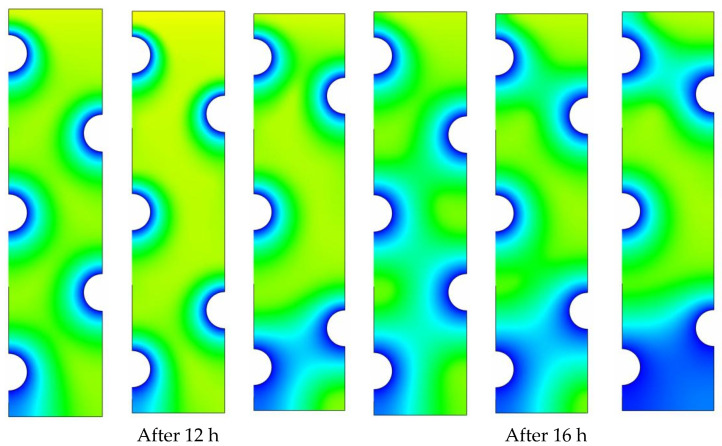
Contours of the PCM temperature progression for three different HTF-tube arrangements of P5, p5-nu-1, and p5-nu-2 over multiple solidification durations.

Figure 11 shows the temperature contour distributions for the three tube configurations P5, P5-nu-1, and P5-nu-2 during the periods (t = 4, 8, 12, and 16 h). As time goes on, a noticeable shift in the color of the isotherms is seen when the HTF tubes are shifted away from their original position in the reference case of P5. As stated in the earlier sections, there are gradual temperature gradients caused by the buoyancy-driven flow generated in the upper regions of the domain, which causes a nonuniformity in the color of the isotherms when comparing the relatively larger spaced tube cases (P5-nu-1) and (P5-nu-2) with the base case (P5). It can be concluded based on the aforementioned isotherm discoloration that natural convection plays a significant role in the heat removal process, which helps the solidification process. However, repositioning the HTF tubes to the top places in the cases of P5-nu-1 and P5-nu-2 reduces the convection contribution. This is due to the fact that moving HTF tubes to higher positions raises the local flow resistance, which reduces the convection-assisted circulation of liquid PCM. Therefore, repositioning the HTF tubes in the right vertical direction is not recommended during the energy discharging mode. Data from Table 7 shows that the PCM can solidify at an energy removal rate of 99.42 W in the base case of P5, but this rate decreases to 98.33 and 95.01 W when the HTF tubes are moved vertically on the left side in the cases of p5-nu-1 and p5-nu-2, respectively. Therefore, repositioning of the HTF tubes on the left side of the unit negatively reduces the energy removal rate by about 1.2 and 4.4%, respectively.

### 5.4. Impact of Varying the HTF Temperature

**Figure 12:**
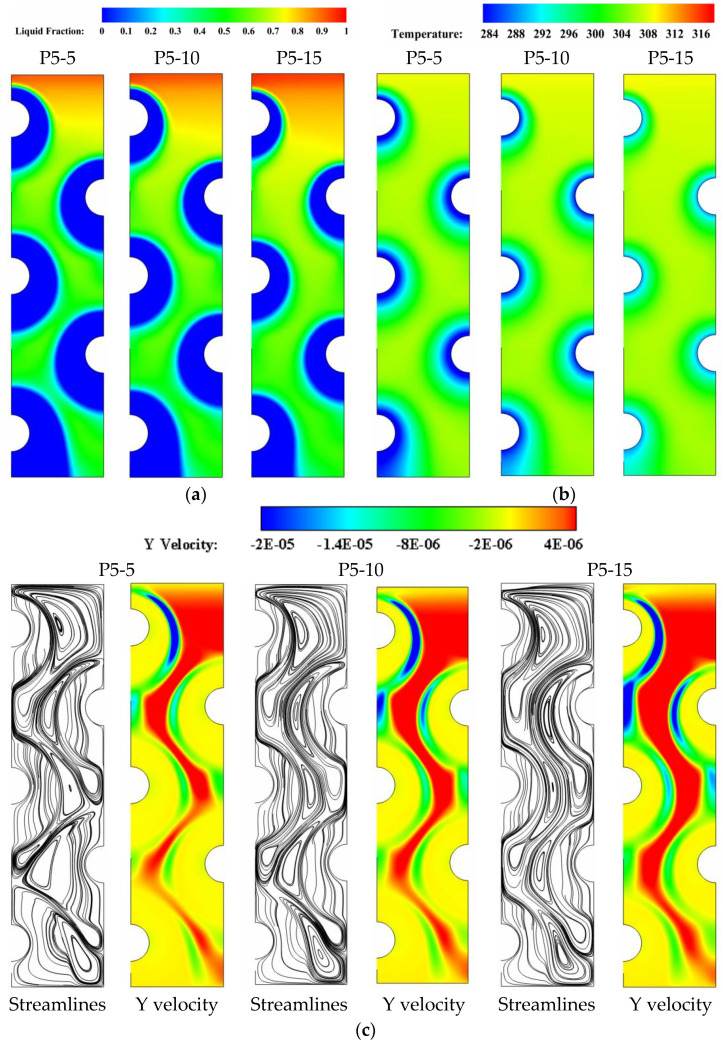
(**a**) liquid-fraction contours, (**b**) isotherms, and (**c**) velocity field and streamlines for the cases of P5-5, P5-10, and P5-15 after 8 h of solidification.

To explore the effect of HTF temperature on facilitating faster solidification rates of PCM in the multi-tube storage unit, the base case of uniformly spaced tubes (P5) is investigated with three HTF temperatures of 5, 10, and 15 °C so that the new cases are referred to P5-5, P5-10, and P5-15. Figure 12a–c depicts the liquid-fraction contours, isotherms, streamlines and velocity fields for the cases of P5-5, P5-10, and P5-15, respectively, during the solidification period (t = 8 h). Data from these figures reveals that the values of liquid fraction drop linearly as greater HTF temperatures are applied. This can be seen in Figure 12a when the sizes of blue zones, which represent the solidified PCM layers, are compared between the considered cases P5-5, P5-10, and P5-15. Meanwhile, raising the HTF temperature from 5 °C in the case of P5-5 to 15 °C in the case of P5-15 results in a significant shift in the color of isotherms, indicating higher values of PCM temperatures. Additionally, increasing the HTF temperature from P5-5 to P5-15 can cause the velocity field to show decreased velocities of liquid PCM, with a noticeable shrink in the red layers that represent zones of high velocities. In addition, applying higher HTF temperatures can trigger the streamlines to form smaller recirculation cells, which is a sign of a lower convective contribution to the heat transfer process during the energy discharging mode. After 16 h of solidification, the results indicate that the liquid fraction is 0.035, 0.078, and 0.16 for the cases of P5-5, P5-10, and P5-15, respectively. This means that increasing the HTF temperature causes a reduction in the cooling effect on the PCM side, resulting in slower solidification rates on that side.

**Figure 13:**
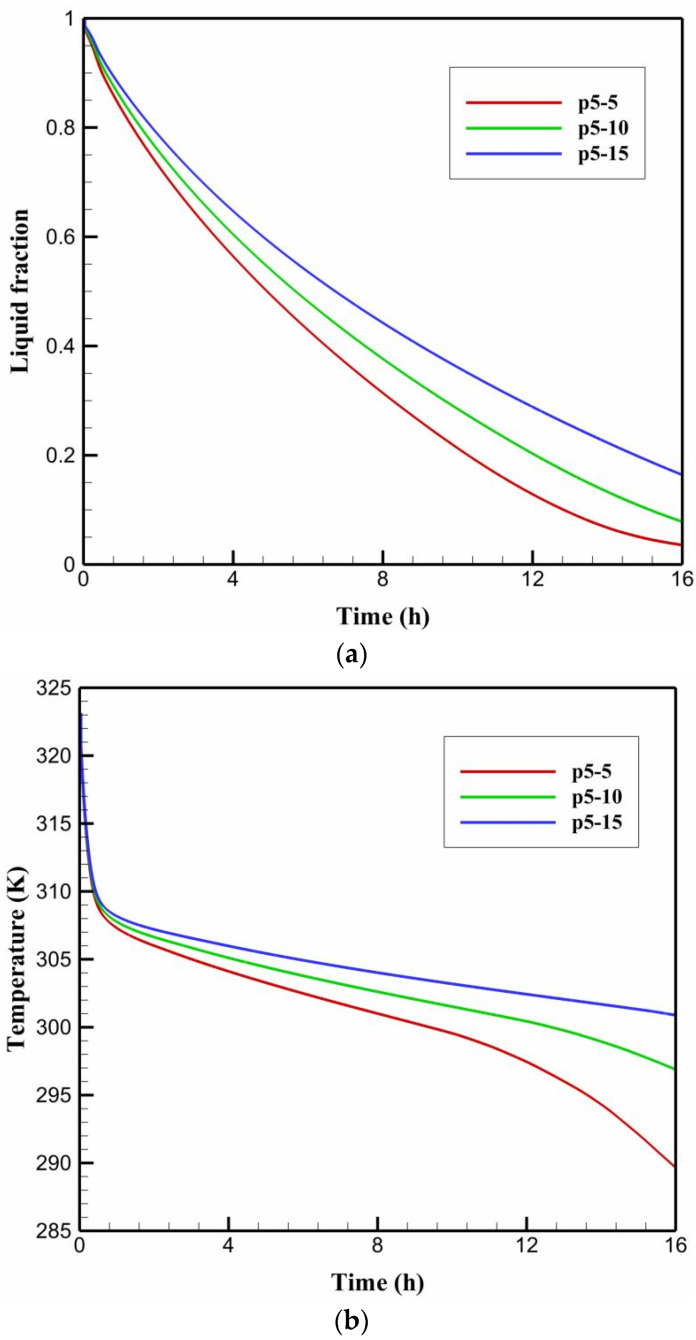
The time-wise variation of (**a**) liquid-fraction profile, and (**b**) average temperature for the PCM solidification with the cases of P5-5, P5-10, and P5-15.

The impacts of varying the HTF temperature on the time-wise evolution of the liquid-fraction and the average temperature profiles are depicted in Figure 13a,b respectively. The data from these figures indicate that increasing the HTF temperature from the case P5-5 of 5 °C to the case P5-15 of 15 °C negatively impacts the PCM liquid-fraction and average temperature behaviors. It can be observed that that the liquid-fraction and average temperature behavior curves in the case of P5-5 preserve the lowest values, resulting in a shorter time required for discharging (solidification time) as compared to the other cases under discussion. The data from Table 8 indicates that the PCM solidifies at an energy removal rate of 107.12 W at HTF temperature of 5 °C, but this rate decreases to 99.42 and 89.77 W when the HTF temperature increases to 10 and 15 °C, respectively. Therefore, decreasing the HTF temperature from 15 °C to 10 and 5 °C can increase the heat removal rate by around 7 and 16%, respectively.

## 6. Conclusions

A design model of a TES unit cooled by HTF tubes was numerically evaluated. The geometry of LHTES included different numbers of HTF tubes distributed in different positions in the unit for various cases. The effect of tubes’ locations on the discharging procedure in the TES unit was described using the growth of the solid phase, discharging maps, streamlines, and temperature distribution. It should be noted that such an improvement is achieved without additional cost, as no additional material should be used in the fabrication of an LHTES unit. Several facts could be indicated from this study:

- The optimal number of HTF tubes is employed, natural convection will be able to contribute more effectively to the energy removal process.
- The values of liquid fraction after 16 h of solidification are 0.078, 0.075, and 0.090 for the cases of P5, P5-u-1, and P5-u-2, respectively.
- Moving the HTF tubes to medium positions along the vertical direction is relatively better for enhancing the solidification of PCM with multiple HTF tubes.
- Replacing the HTF tubes on the left side of the unit can slightly improve the energy removal rate by about 0.2 in the case of p5-u-1 and decreases by 1.6% in the case of p5-u-2.
- This means that increasing the distance between the tubes in the vertical direction has a detrimental effect on the PCM solidification mode.
- Repositioning of the HTF tubes on the left side of the unit negatively reduces the energy removal rate by about 1.2 and 4.4%, respectively.
- Increasing the HTF temperature causes a reduction in the cooling effect on the PCM side, resulting in slower solidification rates on that side.
- Decreasing the HTF temperature from 15 °C to 10 and 5 °C can increase the energy removal rate by around 7 and 16%, respectively.

The present study confirms that the arrangement of the channels, which improves the solidification process and concentrates on the free convection impact in LHTES should be considered in this field. The spacing between the tubes could be an extra important designing factor, as it basically controls the solidified regions and the dominates of general stream flows. The study of using many tiny diameter channels with different distributions could be a topic for future studies.

## Figures and Tables

**Table 1:** Thermophysical properties of RT35 [61].

Property	RT35
Specific heat (kJ/kgK)	2
Viscosity (Pas)	0.023
Heat of fusion (kJ/kg)	170
Liquidus temperature (°C)	35
Density (kg/m^3^)	815
Thermal conductivity (W/mK)	0.2
Solidus temperature (°C)	29
Thermal expansion coefficient (1/K)	0.0006

**Table 2:** The analysis of grid and time step sizes.

Number of nodes	35,122	52,682	**52,682**	52,682	10,5364
Time step size	0.2	0.1	**0.2**	0.4	0.2
Solidification rate (W)	53.43	53.01	**52.89**	52. 55	52.74

**Table 3:** The average PCM temperature (K) in both the present study and the work of Al-Abidi et al. [65] with a calculated error.

Time (s)	PCM Average Temperature (K) (Present Study)	PCM Average Temperature (K) (Al-Abidi et al. [65])	Error
0	364	364	-
5	351.5	351.8	0.0085%
10	348.8	349.2	0.011%
15	347.3	348	0.02%
20	345	346	0.029%
25	344	345	0.029%
30	343.2	344	0.02%
35	342.6	343.1	0.0146%

**Table 4:** The PCM liquid-fraction progression for three different HTF-tube arrangements of P3, P5, and P7 over multiple solidification durations.

	L.F (4 h)	L.F (8 h)	L.F (12 h)	L.F (16 h)
P3	0.9	0.68	0.41	0.22
P5	0.84	0.62	0.34	0.075
P7	0.86	0.65	0.38	0.1

**Table 5:** The energy removal rate during solidification in the cases of P5, p5-u-1, and p5-u-2.

Cases	P5	P5-u-1	P5-u-2
Energy density rate during solidification (watt)	99.42	99.54	97.94

**Table 6:** The PCM liquid-fraction progression for three different HTF-tube arrangements of P5, p5-nu-1, and p5-nu-2 over multiple solidification durations.

	L.F (4 h)	L.F (8 h)	L.F (12 h)	L.F (16 h)
P5	0.84	0.62	0.34	0.075
P5-nu-1	0.81	0.57	0.27	0.078
P5-nu-2	0.79	0.53	0.26	0.09

**Table 7:** Heat removal rate during solidification in the cases of P5, p5-nu-1, and p5-nu-2.

Cases	P5	P5-u-1	P5-u-2
Average energy removal rate during solidification (watt)	99.42	99.54	97.94

**Table 8:** The energy removal rate during solidification in the cases of P5-5, P5-10, and P5-15.

Cases	P5-5	P5-10	P5-15
Average energy removal rate during solidification (watt)	107.12	99.42	89.77

## Data Availability

Not applicable.

## Nomenclature

$A_{m}$	The mushy zone constant	$t_{m}$	solidification time (s)
*C*	Inertial coefficient	T	Temperature (K)
$C_{p}$	PCM specific heat (J/kgK)	$T_{m}$	Melting point temperature (K)
*D*	Diameter (mm)	$\vec{V}$	Velocity vector (m/s)
g	Gravitational acceleration (m/s^2^)		
*H*	Total Enthalpy (kJ/kg)	Greek symbols	
*h*	Enthalpy (kJ/Kg)	β	Thermal expansion coefficient (1/K)
K	thermal conductivity (W/mK)	λ	Liquid fraction
L	Latent heat of fusion (J/kg)	μ	Dynamic viscosity (kg/ms)
m	PCM mass (kg)	ρ	Density (kg/m^3^)
P	Pressure (Pa)		
$\dot{Q}$	Solidification rate (J)		
